# An expert perspective on diversity-oriented standards for assessing sex and gender in clinical research

**DOI:** 10.3389/fpsyt.2024.1448487

**Published:** 2025-01-29

**Authors:** Hannah R. Hambruch, Nora M. Laskowski, Robert-Paul Juster, Georg Halbeisen, Georgios Paslakis

**Affiliations:** ^1^ University Clinic for Psychosomatic Medicine and Psychotherapy, Medical Faculty, Ruhr-University Bochum, Luebbecke, Germany; ^2^ Research Center of the Institut Universitaire en Santé Mentale de Montréal, Department of Psychiatry and Addiction, Université de Montréal, Montréal, QC, Canada

**Keywords:** expert study, diversity, guidelines, LGTB+, LGBTQ+, gender, survey

## Abstract

**Introduction:**

Randomized controlled trials require diverse patient groups to ensure broad applicability of results. However, gender minorities are often not included, which affects the generalizability and equity of healthcare outcomes. Inclusive research must consider the diversity of sex and gender to eliminate inequalities and improve health outcomes.

**Methods:**

A two-stage expert survey was conducted using a self-developed questionnaire in which the constructs of sex, gender, and gender expression were considered. Experts rated the importance and practicality of assessing these concepts in clinical trials and evaluated terms for suitability and comprehension. In addition, existing definitions were refined. Consensus was defined as 70% agreement or disagreement.

**Results:**

14 out of 17 participating experts agreed on the importance to independently assess sex assigned at birth, and 9 out of 16 emphasized this for gender identity in clinical trials. Sex should be assessed with “Please specify your sex assigned at birth” and the answer categories “female”, “male”, “intersex”. Gender identity should be assessed with “I identify as…” and the answer categories “woman”, “man”, “nonbinary”, “trans woman”, “trans man”, “genderqueer”, “genderfluid”, “agender”, “two spirit”. Assessment of gender expression depends on the research question and may not be relevant for every study.

**Discussion:**

Our findings emphasize inclusivity by providing multiple gender options and improve data accuracy by allowing individuals to accurately report their gender identity. The results emphasize the importance of distinguishing between sex assigned at birth, gender identity, and gender expression in research. This ensures that gender diversity is accurately represented and considered, improving the relevance and inclusivity of clinical trials.

## 1 Introduction

Randomized controlled trials (RCTs) are considered the gold standard in clinical research for investigating the course and progression of diseases and establishing treatment efficacy (1, 2). They represent an opportunity to improve both the health and healthcare of people worldwide. In each clinical study, data are collected from a particular group of patients with a specific focus on interventions. The patient selection significantly impacts the quality of the data obtained. For clinical studies to be as broadly applicable as possible to a large patient population, studies must be designed in a manner that allows their results to be extrapolated to a diverse patient cohort (3).

There is increasing evidence indicating that different subgroups of patients respond differently to treatment. Moreover, disease courses may vary among distinct patient groups due to factors such as sex, genetic background, age, and race (4, 5) to name but a few factors. Examples of such varying treatment responses and disease occurrences include the observation of higher levels of some of the commonly used antiretroviral agents in the treatment of the human immunodeficiency virus (HIV) in diverse groups, which can lead to higher concentrations and thus improved efficacy, but in other cases be linked to increased adverse events (6). Another example is the evidence showing that transgender adolescents have a higher risk of suicidality compared to cisgender adolescents (7–9).

If medical research aims to produce results that can foster efficient, equitable, and safe healthcare for all, underrepresented groups, such as sexual and/or gender minorities (SGM), must also be represented in clinical studies. On the one hand, awareness concerning the importance of various social and diversity-related determinants of health is increasing in global research (10, 11). At the same time, research shows that certain groups are still underrepresented in clinical studies (12–15). For instance, disparities in the occurrence and distribution of brain tumors among various ethnic groups suggests distinct genetic and hereditary influences on tumor development that clinical trials often fail to include by not treating race as a reported factor. As shown by Taha et al. (15), only 27% of published drug- and biological-based clinical trials reported on race and/or ethnicity in an analysis of North American brain tumor studies. Moreover, clinical drug trials continue to lack female research participants, who account for only one third of participants in clinical trials published (16). This highlights that current issues in diversity-sensitive research that not only lacks of representation of certain cohorts but also uses inconsistent assessment of diversity-specific characteristics.

There has been some progress in designing more inclusive trials regarding characteristics such as race and ethnicity (17, 18). Concerning health determinants such as sex and gender, the launch of the “Sex and Gender Equity in Research” (SAGER) guidelines in 2016 was a significant step in encouraging the systematic reporting of sex and gender in health research (19). Sex is defined as “based on external genitalia, with consideration given to other factors in cases of ambiguity” (20), while gender refers to “a person’s internal sense of their identity of being a boy, a man, or male, a girl, a woman, or female, or an alternative gender such as genderqueer, gender nonconforming, gender neutral, that may or may not align with the sex assigned at birth or secondary sex characteristics” (20). Today, it is recommended to use “male/female/intersex” in reference to sex and boy/girl or man/woman or gender diverse person in reference to gender. While there is some evidence regarding differences in disease pathomechanisms, disease manifestation, and treatment responses that may be attributed to sex (assigned at birth), the incorporation of aspects around gender may add an important complementary dimension to understanding variability in clinical presentation and therapy outcomes – especially in social sciences, thus in areas addressing psychological and socio-cultural influences on health and disease.

Most scientific journals still lack reporting guidelines regarding sex and gender diversity (21). In addition, sex and gender-based analyses are inadequately investigated in medical research, as evidenced by a text-mining analysis of 8,836 articles across nine clinical subspecialties where all disciplines – with the exception of cardiology – demonstrated an underrepresentation (less than 20% distribution) of research about gender differences in clinical management (22). Despite increasing awareness of the importance of implementing sex and gender into health research, uncertainty persists regarding the definition and use for these concepts. Sex and gender are often used interchangeably in scientific papers (23–25), although they are distinct and non-interchangeable terms (26). Conventional approaches describing clinical trial populations solely based on sex assigned at birth may overlook factors linked to disease risk.

Beyond sex and gender considerations, members of the LGBTQIA+ (lesbian, gay, bisexual, transgender, queer, intersex, asexual) community continue to be overlooked in trial designs. Consider the following: transgender patients have four times higher rates of receiving a mental health diagnosis than cisgender patients (27). Due to the higher exposure to stigma and prevalence of mental health concerns compared to cisgender individuals (27–29), transgender individuals represent a population susceptible to adverse mental health. The perceived or actual social stigma and discrimination these individuals experience may significantly impact their willingness and ability to access appropriate medical care, constituting a critical barrier to healthcare (30, 31). Additionally, their experienced social stigma is associated with psychological distress (28). Therefore, a binary measurement of sex or gender excludes transgender and intersex individuals and misses the opportunity to develop health measures for a gender-diverse patient group (29) with unique health and wellness needs.

Notably, there are variations in the dimensions of sex, gender, and sexual orientation that different sex/gender measures are able to capture. Existing measures, such as the two-step method (32), capture three dimensions of sex and gender: sex assigned at birth, current gender identity, and transgender status (through cross-classification), and have been rated as effective and easy to identify transgender participants in population studies (32–36). Despite this, when employing two-step gender measures, several challenges may arise, including the lack of collecting additional dimensions of gender [e.g., gender expression, which “refers to how an individual communicates their gender through behaviors, attire, communication styles, and interests, within the context of their culture” (37)] and/or inadequate response options for adolescents who are gender nonconforming or questioning (36) as well as sexual orientation variables.

Growing evidence underscores the relevance of gender expression as a health determinant; however, data on this topic remain scarce, representing untapped potential for advancing population health (38). Researchers emphasize that incorporating measures of gender expression into surveys is essential for capturing the diverse ways people experience gender and for understanding how gender inequality influences life opportunities and health outcomes (39, 40). Gender expression can vary over time and context and is especially critical as an emerging health determinant for children (38, 41, 42). For example, Gordon et al. (43) identified gender expression as a risk indicator for disordered weight control behaviors, particularly among adolescent boys perceived as more feminine, who may face stigma for defying societal gender norms. Some studies link feminine behaviors to better health outcomes and masculine behaviors to poorer health (44). The intersection of gender expression and identity is vital for health; masculinity has been associated with better self-rated health in cisgender men, while femininity has been linked to better self-rated health in cisgender women (40). As another example, Samulowitz et al. (45) demonstrated that gendered norms shape not only how men and women experience and express pain but also how healthcare providers respond to it. Thus, to enhance understanding of the health implications tied to gender expression and identity, inclusive and comprehensive measures are essential in research.

One solution involves incorporating additional response options such as genderqueer and other relevant categories, or using multidimensional measures of sex and gender (46). Stadler et al. (47) suggest incorporating a “prefer not to answer” choice to offer respondents more flexibility in their responses, refrain from using the term “other”, and to include an open-ended option for individuals to self-identify as they please. Additionally, they suggest utilizing a list containing multiple categories (e.g., nonbinary) to achieve a balance between recognition, inclusivity, and practicality. The remaining challenges when measuring sex, gender, and sexual orientation include determining the number of categories, deciding whether categorical versus dimensional assessments are preferable, and ensuring that the terms used are not only comprehensible to patients but also broadly accepted by the respective individuals identifying as such.

The current inadequacy in the representation of sex, gender, and sexual orientation along with the methods for assessing these parameters highlights the need for further research in this field. The present study aims to examine the understanding and usability of existing definitions through a two-staged online survey involving international experts in the field of sex and gender diversity research. Based on this survey, we present insights on assessing sex, gender, and gender expression in clinical studies that may inform future research and practice, regardless of the specific research question.

## 2 Material and methods

### 2.1 Search strategy and eligibility criteria for experts

As this is a survey of experts, the study was exempted from ethics approval on January 3rd, 2022, by the Ethics Committee of the Medical Faculty of the Ruhr-University Bochum (AZ 2022-883). The study was prospectively registered with AsPredicted under application #12423.

**Figure 1:**
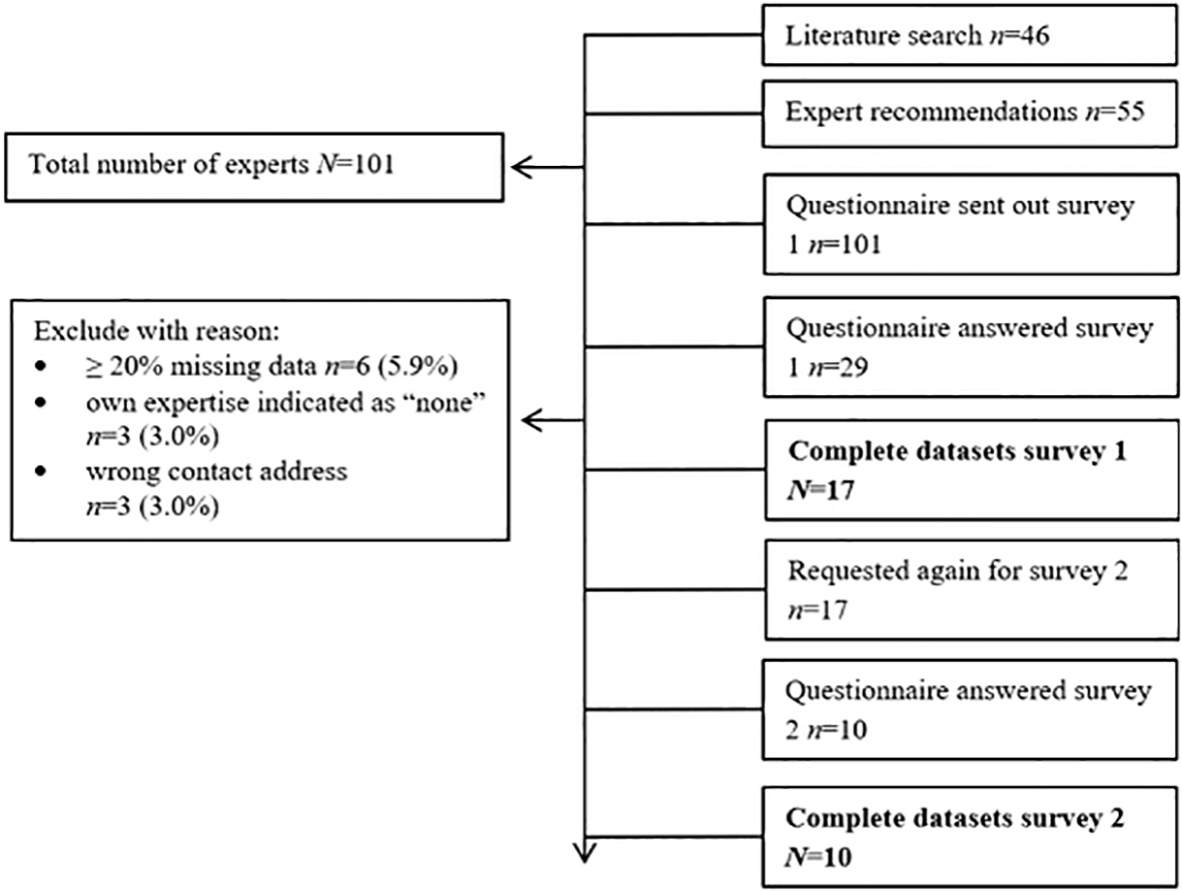
Flow Chart.

A search of published literature was conducted in PubMed using the keywords “gender” or “sex” or “measure(-ment)” and “operationalization” to identify experts in the field. No restrictions were applied regarding journal, time period, or geographic origin of the publication. Ultimately, articles published between 2003 and 2021 were included. The reference lists of included articles were also hand-searched for additional relevant literature, and experts were asked to recommend additional experts who were invited to participate in the survey. In total, 45 experts were initially chosen based on the literature search, and an additional 55 were included (see **Figure 1**). As both the first and last authors were assumed to possess expertise – with the first author leading e.g., data collection and analysis, and the last author guiding e.g., study design, and providing insights as regards contents –the first and last authors of publications on sex and gender diversity in medical research were identified through literature search and invited to participate in the survey.

### 2.2 Questionnaire

The questionnaire used in the survey was developed based on the clinical and scientific expertise of the study team (30, 48, 49), a review of the literature, and the definitions of the American Psychological Association regarding the different concepts under investigation (20). Initially, the first author designed the questionnaire, which was then revised and consented to by the entire study team. This process involved evaluating the strengths and weaknesses of items, and ensuring that instructions, questions, and response options were presented in an inclusive manner.

The questionnaire used in the first survey consisted of five sections, three of them focusing on the concepts of 1) sex, 2) gender, and 3) gender expression. Each part was designed equally and comprised a total of 42 items, resulting in a total of 138 items for the entire questionnaire. The fourth part consisted of a presentation of existing measures, and experts were asked to specify their familiarity with them. The fifth part was used to collect socio-demographic information about the experts.

The definitions, which were first presented for each construct, were sourced from the American Psychological Association (APA) “Guidelines for Psychological Practice with Transgender and Gender Nonconforming People” (20). Experts were asked to indicate their level of agreement with these definitions (“strongly disagree”, “moderately disagree”, “moderately agree”, “strongly agree”) and to suggest any changes or additions. Their feedback was considered and incorporated into the re-evaluated definitions in the second round of the survey. According to the APA guidelines, sex is typically determined at birth based on the external genitalia and may involve other factors in cases of ambiguity. It acknowledges that for transgender and gender nonconforming individuals, there may be discrepancies between biological sex and gender identity. Gender identity is defined as an individual’s internal, deeply felt sense of being boy/man, girl/woman, or another gender identity, which may not be outwardly apparent to others. Gender expression is described as encompassing how an individual communicates their gender through actions and behaviors, such as clothing choices, communication styles, and interests.

After establishing these definitions, experts were tasked with rating the importance and practicality of assessing these concepts in clinical trials using a rating scale (“not at all”, “slightly”, “mostly”, “extremely”). Additionally, they were asked to evaluate the suitability of various terms (e.g., “female”, “trans man”, and “nonbinary”) for assessing these concepts, using a scale ranging from “not suitable” to “very suitable”. Furthermore, experts were prompted to assess the ease of understanding these terms, ranging from “very difficult” to “very easy”.

In the subsequent section of the questionnaire, experts were prompted to specify their familiarity with existing gender assessment tools, categorizing their knowledge as either “known and used”, “known but not used”, or “not known”. These measures included the “two-step method” (32), the “Multidimensional Test Measure” (46), and the “Gender Identity Scale” (50). Following this, the experts were asked to assess the consideration of additional concepts such as “sexual orientation”, “romantic orientation”, and “sexual behaviors” in clinical studies that evaluate sex, gender, and/or gender expression.

To allow experts to identify any overlooked aspects, open-ended questions were incorporated into various sections of the survey (e.g., “please indicate whether you would add any term that describes gender that has not been listed here but should be included”). Additionally, the survey concluded by gathering information on the sociodemographic characteristics of the experts, their proficiency in the English language, and their level of (clinical) experience with gender and sex related research, with response options ranging from “none” (those participants were then excluded) to “a great deal”.

The second round of the questionnaire incorporated questions from the first version of the questionnaire that did not achieve consensus (see section 2.3), along with new questions derived from the feedback and comments provided by the experts (e.g., “I am not Indigenous myself, but I have seen lists that include Two Spirit”), resulting in a total of 115 items. The second questionnaire did not re-query sociodemographic information.

### 2.3 Consensus and multi-step survey

The first questionnaire was sent to 101 experts on March 20^th^, 2023, via an invitation email that included a link to the online survey. A reminder was sent on April 21^st^. A total of 29 questionnaires were answered in the first survey (response rate: 28.7%), which was closed on May 24^th^. We included 17 complete datasets for survey 1 (see **Figure 1** for reasons). The second questionnaire was then revised based on the experts’ comments and consensus analyses and was distributed on July 17^th^, 2023, to all 17 experts with complete datasets. Reminders were sent after four weeks. The second survey was closed on September 8^th^. The response rate in the second survey was 58.8%.

Aligned with other expert studies (e.g., 51–53), the consensus criteria were established at 70% agreement or disagreement for dichotomized responses (e.g., very suitable/suitable and less suitable/not suitable). Items that reached consensus (agreement ≥70%) in the first survey were not included in the second survey and were implemented in the final results. Items that were rejected (disagreement ≥70%) were also removed and not included in the final recommendations. Alongside each item lacking consensus (<70% agreement/>70% disagreement), anonymized group responses from the first survey were provided (e.g., “5.9% answered very suitable, 17.6% suitable, 35.3% less suitable, 41.2% not suitable in the first survey”). Based on these group responses, every expert was asked to re-rate questions that did not reach consensus again and give their opinion on additional questions marked as new (e.g., “New question: Which of the pairs of terms would you prefer when asking about sex assigned at birth?”) that were added based on the experts’ comments.

## 3 Results

### 3.1 Experts

**Figure 1** depicts the expert flow of both survey rounds. The sociodemographic characteristics of the experts (assessed in survey 1) are shown in **Table 1**.

**Table 1:** Sociodemographic variables.

Variables	*N* = 17 *n* (%)
English skills	
Native/Bilingual	9 (52.9)
Full professional proficiency	3 (17.6)
Professional working proficiency	5 (29.4)
Level of expertise/knowledge to gender/sex related research	
A great deal	8 (47.1)
Quite a bit	6 (35.3)
A moderate amount	3 (17.3)
Age; mean *(SD)*	40.1 (10.9)
Community Member	
Yes	11 (68.8)
No	5 (31.3)
Gender (multiple answers possible)	
Ciswoman/Female/Woman	8 (40.0)
Male/Man	5 (25.0)
Genderqueer/Queer	2 (15.0)
Nonbinary	2 (10.0)
No category	1 (5.0)
Two Spirit	1 (5.0)
Area of profession	
Psychology	5 (29.4)
Medicine	2 (11.8)
Public health	3 (17.6)
Other (no specification)	7 (41.2)
Concrete field of work (multiple answers possible)	
Addiction and mental health	1 (5.6)
(Affective) neuroscience/neuroendocrinology/biopsychology	3 (16.7)
Clinical psychology	1 (5.6)
Gender/sexuality/social psychology	3 (16.7)
Sociology	1 (5.6)
Pediatrics	1 (5.6)
Psychiatry	1 (5.6)
(Health equity) research (funding, methods, data)	4 (22.2)
Teaching/Health science education	2 (11.1)
Knowledge translation	1 (5.6)
Country of work	
Canada	9 (52.9)
Germany	1 (5.9)
Switzerland	1 (5.9)
USA	6 (35.3)

### 3.2 Sex assigned at birth

Fourteen out of 17 experts (82.4%) agreed on the importance of independently assessing the sex assigned at birth in clinical trials, regardless of the research question (out of 17 experts: 41.2%, n=7 answered “extremely”; 41.2%, n=7 answered “mostly”; 11.8%, n=2 answered “slightly”, 5.9%, n=1 answered “not at all”).

The definition of the APA (20) underwent adjustments in the first step based on comments received and was then re-evaluated in the second survey. Consensus was reached by eight out of ten experts (80%) on the acceptability of the revised definition for sex assigned at birth:

“Sex assigned at birth may be seen as an epistemological construction and a form of classification that assumes a binary since it is mostly based on the appearance of the external genitalia. The term “sex assigned at birth” may also be misleading, as sex is often identified during pregnancy, recognized at birth and then entered accordingly as legal sex in legal documents (e.g., birth certificate). A recognition of sex at birth that is based on the appearance of the external genitalia only, disregards the fact that sex is multifaceted and that next to organ-based sex (internal and external organ development), also chromosome-based sex (presence or absence of the SRY region) and endocrinological sex (relative proportion of sex hormones levels) and other biological sex-based factors may depict categories to categorize sex beyond the binary. When the external genitalia appear ambiguous in regard to the usual binary phenotypes of male or female and/or there is an incongruence between the recognition of external genitalia and other sex-related aspects (e.g., internal genitalia, chromosomes), individuals are considered intersex or people with variations in sexual characteristics. For most individuals, sex determination based on the external genitalia and the binary male/female distinction is in congruence with gender identity later in life. Still, it is important to acknowledge intersex conditions, as well as trans* and non-binary/gender non-conforming individuals whose gender identity varies from the sex attributed to them at birth.”

At this juncture, it is important to note that some experts have remarked, in response to the revised definition, that it remains too complex and may need further simplification, if deemed necessary.

There was no consensus on how sex assigned at birth should be assessed in either the first survey (out of 16 experts: 43.8%, n=7 answered “categorically”; 37.5%, n=6 answered “dimensionally”; 18.8%, n=3 answered “other”) or the second survey (out of ten experts: 50.0%, n=5 answered “categorically”; 30.0%, n=3 answered “multi-dimensionally”; 20.0%, n=2 answered “with an open field”). However, there was a consensus that sex assigned at birth should be collected with the statement “Please specify your sex” (nine out of ten experts; 90.0% consensus in the second survey). **Table 2** presents the results on the suitability and understandability of terms for the assessment of sex assigned at birth.

**Table 2:** Suitability and understandability of terms for the assessment of sex assigned at birth.

*How suitable are the following terms to assess the sex of patients or participants in clinical and other research studies?*								
Terms	First survey				Second survey			
	Answer Scale							
	Very suitable n (%)	Suitable n (%)	Less suitable n (%)	Not suitable n (%)	Very suitable n (%)	Suitable n (%)	Less suitable n (%)	Not suitable n (%)
**Female^a^**	13 (76.6)	4 (23.5)	–	–	–	–	–	–
**Male^a^**	13 (76.6)	4 (23.5)	–	–	–	–	–	–
**Intersex^a^**	11 (64.7)	4 (23.5)	2 (11.8)	–	–	–	–	–
**Diverse^a^**	3 (17.6)	1 (5.9)	4 (23.5)	9 (52.9)	–	–	–	–
**Woman^b^**	1 (5.9)	6 (35.3)	2 (11.8)	8 (47.1)	1 (10.0)	–	1 (10.0)	8 (80.0)
**Man^b^**	1 (5.9)	6 (35.3)	2 (11.8)	8 (47.1)	1 (10.0)	–	1 (10.0)	8 (80.0)
**Transgender^a^**	2 (11.8)	1 (5.9)	4 (23.5)	10 (58.8)	–	–	–	–
**Trans woman^a^**	2 (11.8)	1 (5.9)	4 (23.5)	10 (58.8)	–	–	–	–
**Trans man^a^**	2 (11.8)	1 (5.9)	4 (23.5)	10 (58.8)	–	–	–	–
**Trans-masculine^a^**	2 (11.8)	2 (11.8)	2 (11.8)	11 (64.7)	–	–	–	–
**Trans-feminine^a^**	2 (11.8)	2 (11.8)	2 (11.8)	11 (64.7)	–	–	–	–
**Nonbinary^a^**	3 (17.6)	2 (11.8)	2 (11.8)	10 (58.8)	–	–	–	–
**Genderqueer^a^**	2 (11.8)	1 (5.9)	3 (17.6)	11 (64.7)	–	–	–	–
**Genderfluid^a^**	2 (11.8)	1 (5.9)	3 (17.6)	11 (64.7)	–	–	–	–
**Agender^a^**	2 (11.8)	1 (5.9)	3 (17.6)	11 (64.7)	–	–	–	–
**Something other than male or female^b^**	2 (11.8)	4 (23.5)	3 (17.6)	8 (47.1)	–	1 (10.0)	2 (20.0)	7 (70.0)
**Sometimes male, sometimes female^a^**	1 (5.9)	–	3 (17.6)	13 (76.5)	–	–	–	–
Person with vagina^b,c^	–	–	–	–	3 (30.0)	1 (10.0)	1 (10.0)	5 (50.0)
Person with uterus^b,c^	–	–	–	–	3 (30.0)	2 (20.0)	–	5 (50.0)
Person with penis^b,c^	–	–	–	–	3 (30.0)	1 (10.0)	1 (10.0)	5 (50.0)
*How easy to understand are the following terms when assessing sex?*								
	Answer Scale							
	Very easy n (%)	Moderately easy n (%)	Moderately difficult n (%)	Very difficult n (%)	Very easy n (%)	Moderately easy n (%)	Moderately difficult n (%)	Very difficult n (%)
**Female^a^**	15 (88.2)	1 (5.9)	1 (5.9)	–	–	–	–	–
**Male^a^**	15 (88.2)	1 (5.9)	1 (5.9)	–	–	–	–	–
**Intersex^b^**	4 (23.5)	6 (35.3)	6 (35.3)	1 (5.9)	4 (40.0)	3 (30.0)	3 (30.0)	–
**Diverse^a^**	–	2 (11.8)	7 (41.2)	8 (47.1)	–	–	–	–
**Woman^a^**	8 (47.1)	4 (23.5)	3 (17.6)	2 (11.8)	–	–	–	–
**Man^a^**	8 (47.1)	4 (23.5)	3 (17.6)	2 (11.8)	–	–	–	–
Transgender	3 (17.6)	5 (29.4)	6 (35.3)	3 (17.6)	1 (10.0)	4 (40.0)	1 (10.0)	4 (40.0)
**Trans woman^b^**	2 (11.8)	4 (23.5)	8 (47.1)	3 (17.6)	1 (10.0)	6 (60.0)	2 (20.0)	1 (10.0)
**Trans man^a^**	2 (11.8)	3 (17.6)	9 (52.9)	3 (17.6)	–	–	–	–
**Trans-masculine^a^**	1 (5.9)	1 (5.9)	11 (64.7)	4 (23.5)	–	–	–	–
**Trans-feminine^a^**	1 (5.9)	1 (5.9)	11 (64.7)	4 (23.5)	–	–	–	–
**Nonbinary^b^**	–	6 (35.3)	6 (35.3)	5 (29.4)	1 (10.0)	1 (10.0)	4 (40.0)	4 (40.0)
**Genderqueer^a^**	–	1 (5.9)	9 (52.9)	7 (41.2)	–	–	–	–
**Genderfluid^a^**	–	–	9 (52.9)	8 (47.1)	–	–	–	–
**Agender^a^**	–	1 (5.9)	8 (47.1)	8 (47.1)	–	–	–	–
Something other than male or female	2 (11.8)	5 (29.4)	5 (29.4)	5 (29.4)	2 (20.0)	3 (30.0)	4 (40.0)	1 (10.0)
**Sometimes male, sometimes female^a^**	1 (6.3)	2 (12.5)	7 (43.8)	6 (37.5)	–	–	–	–

Terms in bold achieved a consensus of ≥70% in one of the two surveys, ^a^terms reached a consensus in survey 1 and were not queried again, ^b^terms reached a consensus in survey 2, ^c^terms were requested by the experts and were newly introduced in the 2^nd^ survey.

### 3.3 Gender (identity)

The experts agreed in their majority (14 out of 16 experts, 87.6%) that it is important to assess the gender (identity) of participants in clinical trials independently of the research question (out of 16 experts: 56.3%, n=9 answered “extremely”; 31.3%, n=5 answered “mostly”; 12.5%, n=2 answered “slightly”). The revision of the definition of gender (identity) of the APA (20) received approval from nine out of ten experts (90%):

“Gender identity may be seen as a social construct describing a person’s internal sense of self that is not necessarily visible to others, may be subject to change over time and lie beyond the man/woman binary, thus corresponding or not to an individual’s sex at birth. Cisgender refers to people for whom their sex assigned at birth corresponds with their gender identity. Transgender or trans* and/or nonbinary people refer to those for whom sex assigned at birth does not correspond with their gender identity or a binary conceptualization of gender identity. Gender is a broader social and cultural construct that does not only include gender identity (self-identification) but also other aspects such as gender norms, gender relations, gender roles, and gender stereotypes operate across society at the intersection of other systems of hierarchical power such as race and class.”

There was no agreement on the question of how gender (identity) should be assessed in either the first (out of 16 experts: 25.0%, n=4 answered “categorically”; 43.8%, n=7 answered “dimensionally”; 31.3%, n=5 answered “other”) or the second survey (out of ten experts: 10.0%, n=1 answered “categorically”; 20.0%, n=2 answered “multi-dimensionally”; 20.0%, n=2 answered “with an open field”; 40.0%, n=4 answered “multiple-step questions”; 10.0%, n=1 answered “with a mix of categories”). However, there was a consensus that gender should be collected with the statement “I identify as…?” (seven out of ten experts, 70.0% consensus in the second survey). **Table 3** shows the results on the suitability and understandability of terms for the assessment of gender (identity).

**Table 3:** Suitability and understandability of terms for the assessment of gender (identity).

*How important is assessing the gender of patients or participants in clinical research independent of the research questions?*								
Terms	First survey				Second survey			
	Answer Scale							
	Very suitable n (%)	Suitable n (%)	Less suitable n (%)	Not suitable n (%)	Very suitable n (%)	Suitable n (%)	Less suitable n (%)	Not suitable n (%)
**Female^a^**	1 (5.9)	3 (17.6)	6 (35.3)	7 (41.2)	–	–	–	–
**Male^a^**	1 (5.9)	3 (17.6)	6 (35.3)	7 (41.2)	–	–	–	–
**Intersex^a^**	1 (5.9)	1 (5.9)	4 (23.5)	11 (64.7)	–	–	–	–
Diverse	5 (29.4)	4 (23.5)	4 (23.5)	4 (23.5)	1 (10.0)	4 (40.0)	2 (20.0)	3 (30.0)
**Woman^a^**	12 (70.6)	5 (29.4)	–	–	–	–	–	–
**Man^a^**	12 (70.6)	5 (29.4)	–	–	–	–	–	–
**Transgender^b^**	8 (47.1)	3 (17.6)	4 (23.5)	2 (11.8)	3 (30.0)	4 (40.0)	1 (10.0)	2 (20.0)
**Trans woman^a^**	8 (47.1)	9 (52.9)	–	–	–	–	–	–
**Trans man^a^**	8 (47.1)	9 (52.9)	–	–	–	–	–	–
**Trans-masculine^a^**	5 (29.4)	8 (47.1)	4 (23.5)	–	–	–	–	–
**Trans-feminine^a^**	5 (29.4)	8 (47.1)	4 (23.5)	–	–	–	–	–
**Nonbinary^a^**	11 (64.7)	4 (23.5)	2 (11.8)	–	–	–	–	–
**Genderqueer^a^**	10 (58.8)	5 (29.4)	2 (11.8)	–	–	–	–	–
**Genderfluid^a^**	10 (58.8)	5 (29.4)	2 (11.8)	–	–	–	–	–
**Agender^a^**	10 (58.8)	4 (23.5)	3 (17.6)	–	–	–	–	–
**Something other than male or female^a^**	1 (5.9)	2 (11.8)	6 (35.3)	8 (47.1)	–	–	–	–
**Sometimes male, sometimes female^a^**	1 (5.9)	4 (23.5)	5 (29.4)	7 (41.2)	–	–	–	–
**Two spirit^b,c^**	–	–	–	–	6 (60.0)	2 (20.0)	–	2 (20.0)
Cis man^c^	–	–	–	–	4 (40.0)	2 (20.0)	3 (30.0)	1 (10.0)
Cis woman^c^	–	–	–	–	4 (40.0)	2 (20.0)	3 (30.0)	1 (10.0)
No identification^c^	–	–	–	–	3 (30.0)	3 (30.0)	2 (20.0)	2 (20.0)
*How easy to understand are the following terms when assessing gender?*								
	Answer Scale							
	Very easy n (%)	Moderately easy n (%)	Moderately difficult n (%)	Very difficult n (%)	Very easy n (%)	Moderately easy n (%)	Moderately difficult n (%)	Very difficult n (%)
Female	3 (17.6)	7 (41.2)	3 (17.6)	4 (23.5)	2 (20.0)	3 (30.0)	3 (30.0)	2 (20.0)
Male	3 (17.6)	7 (41.2)	3 (17.6)	4 (23.5)	2 (20.0)	3 (30.0)	3 (30.0)	2 (20.0)
**Intersex^a^**	–	4 (23.5)	6 (35.3)	7 (41.2)	–	–	–	–
Diverse	2 (11.8)	5 (29.4)	5 (29.4)	5 (29.4)	–	5 (50.0)	2 (20.0)	3 (30.0)
**Woman^a^**	12 (70.6)	4 (23.5)	1 (5.9)	–	–	–	–	–
**Man^a^**	12 (70.6)	4 (23.5)	1 (5.9)	–	–	–	–	–
**Transgender^b^**	4 (23.5)	5 (29.4)	7 (41.2)	1 (5.9)	2 (20.0)	5 (50.0)	2 (20.0)	1 (10.0)
**Trans woman^a^**	7 (41.2)	7 (41.2)	2 (11.8)	1 (5.9)	–	–	–	–
**Trans man^a^**	7 (41.2)	7 (41.2)	2 (11.8)	1 (5.9)	–	–	–	–
**Trans-masculine^b^**	3 (17.6)	3 (17.6)	9 (52.9)	2 (11.8)	5 (50.0)	3 (30.0)	1 (10.0)	1 (10.0)
**Trans-feminine^b^**	3 (17.6)	3 (17.6)	9 (52.9)	2 (11.8)	5 (50.0)	3 (30.0)	1 (10.0)	1 (10.0)
**Nonbinary^a^**	6 (35.3)	6 (35.3)	4 (23.5)	1 (5.9)	–	–	–	–
**Genderqueer^b^**	3 (17.6)	5 (29.4)	8 (47.1)	1 (5.9)	4 (40.0)	3 (30.0)	3 (30.0)	–
Genderfluid	4 (23.5)	3 (17.6)	9 (52.9)	1 (5.9)	4 (40.0)	2 (20.0)	4 (40.0)	–
Agender	4 (23.5)	3 (17.6)	6 (35.3)	4 (23.5)	3 (30.0)	1 (10.0)	5 (50.0)	1 (10.0)
**Something other than male or female^a^**	1 (5.9)	3 (17.6)	5 (29.4)	8 (47.1)	–	–	–	–
**Sometimes male, sometimes female^a^**	2 (11.8)	3 (17.6)	4 (23.5)	8 (47.1)	–	–	–	–

Terms in bold achieved a consensus of >70% in one of the two surveys, ^a^terms reached a consensus in survey 1 and were not queried again, ^b^terms reached a consensus in survey 2, ^c^terms were requested by the experts and newly introduced in the 2^nd^ survey.

In an effort to potentially streamline the numerous suitable terms, umbrella terms were also explored in the second survey, but none achieved consensus as suitable (see **Supplementary Table S1**).

There was agreement that gender (“a person’s internal sense of their identity of being a boy, a man, or male, a girl, a woman, or female, or an alternative gender” (20);) and gender identity (as a social construct describing a person’s internal sense of self that is not necessarily visible to others, may be subject to change over time and lie beyond the man/woman binary) should be regarded as distinct constructs (eight out of ten experts, 80.0% in the second survey). Additionally, nine out of ten experts (90%) concurred that the terms “woman/man” are more fitting than “female/male”, but there was no consensus on whether “cis woman/cis man” are preferable to “women/men” (five out of ten experts, 50.0%, respectively). The experts expressed the view that “trans woman/trans man” (seven out of nine experts, 77.8%) should be employed instead of the term “transgender” in general (two out of nine experts, 22.2%).

### 3.4 Gender expression

The experts were unsure whether the assessment of gender expression of participants in clinical trials is important regardless of the research question (out of 16 experts: 43.8%, n=7 answered “mostly”; 37.5%, n=6 answered “slightly”; 18.8%, n=3 answered “not at all”). The refined definition of gender expression from the APA (20) received 100% approval by all of the ten experts:

“Gender expression refers to the way an individual, intentionally or not, communicates or is perceived as communicating their gender within a given culture, for example, in terms of clothing, communication patterns, and interests. Gender expression implies cultural norms and thus differs across the world. An individual’s gender expression may or may not reflect their gender identity. In addition, gender expression may or may not be consistent with socially specified gender constructs – the latter possibly depicting a factor of stress for the individual who expresses themselves in a gender non-confirming way.”

There was no agreement on how gender expression should be assessed in either the first (out of 16 experts: 12.5%, n=2 answered “categorically”; 56.3%, n=9 answered “dimensionally”; 31.3%, n=5 answered “other”) or the second survey (out of ten experts: 40.0%, n=4 answered “categorically”; 40.0%, n=4 answered “multi-dimensionally”; 20.0%, n=2 answered “with an open field”). Additionally, there was no consensus on how gender expression should be assessed, neither in the first (out of 17 experts: 11.8%, n=2 answered “I identify as…?”; 11.8%, n=2 answered “I describe myself as…”; 35.3%, n=6 answered “I live as…”; 41.2%, n=7 answered “open text field”) nor in the second survey (out of ten experts: 10.0%, n=1 answered “I describe myself as…”; 40.0%, n=4 answered “I express myself as…”; 50.0%, n=5 answered “open text field”). **Table 4** presents the results on the suitability and understandability of terms for the assessment of gender expression.

**Table 4:** Suitability and understandability of terms for the assessment of gender expression.

*How important is assessing the gender of patients or participants in clinical research independent of the research questions?*								
Terms	First survey				Second survey			
	Answer Scale							
	Very suitable n (%)	Suitable n (%)	Less suitable n (%)	Not suitable n (%)	Very suitable n (%)	Suitable n (%)	Less suitable n (%)	Not suitable n (%)
**Female^a^**	1 (5.9)	3 (17.6)	3 (17.6)	10 (58.8)	–	–	–	–
**Male^a^**	1 (5.9)	3 (17.6)	3 (17.6)	10 (58.8)	–	–	–	–
**Intersex^a^**	1 (5.9)	1 (5.9)	1 (5.9)	14 (82.4)	–	–	–	–
**Diverse^b^**	2 (12.5)	4 (25.0)	4 (25.0)	6 (37.5)	2 (20.0)	1 (10.0)	3 (30.0)	4 (40.0)
**Woman^b^**	5 (29.4)	6 (35.3)	2 (11.8)	4 (23.5)	2 (20.0)	1 (10.0)	2 (20.0)	5 (50.0)
**Man^b^**	5 (29.4)	6 (35.3)	2 (11.8)	4 (23.5)	2 (20.0)	1 (10.0)	2 (20.0)	5 (50.0)
**Transgender^b^**	2 (11.8)	5 (29.4)	4 (23.5)	6 (35.3)	–	–	4 (40.0)	6 (60.0)
**Trans woman^b^**	2 (11.8)	8 (47.1)	2 (11.8)	5 (29.4)	1 (10.0)	–	3 (30.0)	6 (60.0)
**Trans man^b^**	2 (11.8)	8 (47.1)	2 (11.8)	5 (29.4)	1 (10.0)	–	3 (30.0)	6 (60.0)
Trans-masculine	2 (11.8)	7 (41.2)	4 (23.5)	4 (23.5)	2 (20.0)	2 (20.0)	2 (20.0)	4 (40.0)
Trans-feminine	2 (11.8)	7 (41.2)	4 (23.5)	4 (23.5)	2 (20.0)	2 (20.0)	2 (20.0)	4 (40.0)
Nonbinary	3 (17.6)	5 (29.4)	6 (35.3)	3 (17.6)	4 (40.0)	3 (30.0)	–	3 (30.0)
Genderqueer	3 (17.6)	7 (41.2)	3 (17.6)	4 (23.5)	–	6 (60.0)	2 (20.0)	2 (20.0)
**Genderfluid ^b^**	3 (17.6)	7 (41.2)	2 (11.8)	5 (29.4)	–	7 (70.0)	1 (10.0)	2 (20.0)
**Agender^b^**	3 (17.6)	5 (29.4)	3 (17.6)	6 (35.3)	1 (10.0)	2 (20.0)	4 (40.0)	3 (30.0)
**Something other than male or female^a^**	2 (11.8)	3 (17.6)	1 (5.9)	11 (64.7)	–	–	–	–
**Sometimes male, sometimes female^a^**	3 (17.6)	2 (11.8)	3 (17.6)	9 (52.9)	–	–	–	–
**Androgynous^b,c^**	–	–	–	–	6 (60.0)	3 (30.0)	–	1 (10.0)
**Mostly feminine^b,c^**	–	–	–	–	7 (70.0)	2 (20.0)	1 (10.0)	–
**Mostly masculine^b,c^**	–	–	–	–	7 (70.0)	2 (20.0)	1 (10.0)	–
**Somewhat feminine^b,c^**	–	–	–	–	7 (70.0)	2 (20.0)	1 (10.0)	–
**Somewhat masculine^b,c^**	–	–	–	–	7 (70.0)	2 (20.0)	1 (10.0)	–
**Neither feminine nor masculine^b,c^**	–	–	–	–	6 (60.0)	2 (20.0)	2 (20.0)	–
*How easy to understand are the following terms when assessing gender?*								
	Answer Scale							
	Very easy n (%)	Moderately easy n (%)	Moderately difficult n (%)	Very difficult n (%)	Very easy n (%)	Moderately easy n (%)	Moderately difficult n (%)	Very difficult n (%)
**Female^a^**	2 (13.3)	2 (13.3)	4 (26.7)	7 (46.7)	–	–	–	–
**Male^a^**	2 (13.3)	2 (13.3)	4 (26.7)	7 (46.7)	–	–	–	–
**Intersex^a^**	–	2 (13.3)	1 (6.7)	12 (80.0)	–	–	–	–
**Diverse^b^**	1 (6.7)	4 (26.7)	3 (20.0)	7 (46.7)	–	2 (20.0)	3 (30.0)	5 (50.0)
**Woman^a^**	6 (40.0)	5 (33.3)	1 (6.7)	3 (20.0)	–	–	–	–
**Man^a^**	6 (40.0)	5 (33.3)	1 (6.7)	3 (20.0)	–	–	–	–
**Transgender^b^**	1 (6.7)	6 (40.0)	1 (6.7)	7 (46.7)	–	1 (10.0)	4 (40.0)	5 (50.0)
**Trans woman^b^**	3 (20.0)	4 (26.7)	4 (26.7)	4 (26.7)	1 (10.0)	1 (10.0)	5 (50.0)	3 (30.0)
**Trans man^b^**	3 (20.0)	4 (26.7)	4 (26.7)	4 (26.7)	1 (10.0)	1 (10.0)	5 (50.0)	3 (30.0)
Trans-masculine	1 (6.7)	5 (33.3)	6 (40.0)	3 (20.0)	1 (10.0)	4 (40.0)	3 (30.0)	2 (20.0)
Trans-feminine	1 (6.7)	5 (33.3)	6 (40.0)	3 (20.0)	1 (10.0)	4 (40.0)	3 (30.0)	2 (20.0)
Nonbinary	2 (13.3)	4 (26.7)	5 (33.3)	4 (26.7)	1 (10.0)	3 (30.0)	5 (50.0)	1 (10.0)
Genderqueer	–	5 (33.3)	6 (40.0)	4 (26.7)	1 (10.0)	4 (40.0)	4 (40.0)	1 (10.0)
Genderfluid	2 (13.3)	4 (26.7)	5 (33.3)	4 (26.7)	1 (10.0)	5 (50.0)	3 (30.0)	1 (10.0)
**Agender^b^**	–	5 (33.3)	3 (20.0)	7 (46.7)	–	2 (20.0)	5 (50.0)	3 (30.0)
**Something other than male or female^a^**	1 (6.7)	2 (13.3)	4 (26.7)	8 (53.3)	–	–	–	–
Sometimes male, sometimes female	1 (6.7)	4 (26.7)	2 (13.3)	(53.3)	1 (10.0)	4 (40.0)	4 (40.0)	1 (10.0)

Terms in bold achieved a consensus of >70% in one of the two surveys, ^a^terms reached a consensus in survey 1 and were not queried again, ^b^terms reached a consensus in survey 2, ^c^terms were requested by the experts and newly introduced in the 2^nd^ survey.

### 3.5 Other dimensions and measures

The experts agreed that studies that assess sex assigned at birth, gender (identity) and/or gender expression should also consider sexual orientation and sexual behavior (out of 15, eleven experts [73.3%] answered “yes” in the first survey, respectively) as well as romantic orientation (out of ten, seven experts [70%] answered “yes” in the second survey).

There was no consensus regarding the degree of familiarity or recommended use of the measures presented (see **Supplementary Table 2**).

### 3.6 Diversity-oriented recommendations for clinical studies

The following provides a summary of the diversity-oriented recommendations for assessing sex, gender, and gender expression in clinical trials based on insights gathered from 17 international experts. While only aspects that reached a consensus are included, it is important to note that those that did not achieve consensus are not featured in this summary. Experts also indicated that studies surveying the constructs of sex (assigned at birth), gender (identity), and/or gender expression should include assessments of sexual orientation, romantic orientation, and sexual behaviors.

#### 3.6.1 Sex (assigned at birth)

In clinical research, experts recommend assessing sex assigned at birth regardless of the research question. Participants should be prompted with the statement “Please specify your sex (assigned at birth)”, followed by the options: “Female”, “Male”, and “Intersex”.

#### 3.6.2 Gender (identity)

In clinical research, experts also recommend assessing gender (identity) irrespective of the research question. Participants should be prompted with the statement “I identify as…”, followed by the options: “Woman”, “man”, “Nonbinary”, “Trans woman”, “Trans man”, “Genderqueer”, “Genderfluid”, “Agender”, and “Two Spirit”.

#### 3.6.3 Gender expression

Based on the experts’ ratings, the assessment of gender expression depends on the research question and may not be relevant for every study. While no specific statement is recommended for assessing gender expression, the following options should be provided, if a categorical assessment is chosen: “Genderfluid”, “Androgynous”, “Mostly masculine”, “Mostly feminine”, “Somewhat masculine”, “Somewhat feminine”, and “Neither feminine nor masculine”. As noted by the experts, gender expression may also be assessed dimensionally, with the opposite poles labeled as “feminine” and “masculine”.

## 4 Discussion

### 4.1 Discussion of main findings

The aim of this study was to refine current definitions and gather insights on assessing sex assigned at birth, gender (identity), and gender expression in clinical studies independently of the research question. Through an expert survey, we sought to formulate recommendations for these diversity-related assessments. To achieve this, a two-staged online survey was conducted with 17 international experts in the field of sex and gender diversity research. The results of the first survey were evaluated based on a consensus criterion (70% as the threshold for agreement/disagreement), and items without consensus were queried again in a second survey that provided the group responses of survey 1. In addition, the second survey included further, new aspects that the experts were able to add in the first survey to ensure that no aspect was left out.

The experts agreed that it is essential to evaluate both sex assigned at birth and gender (identity), regardless of the research question. There was also a consensus that sex assigned at birth should be assessed by asking participants “please specify your sex” using the following categories: “male”, “female” and “intersex”. Furthermore, there was a consensus that gender (identity) should be assessed by using the statement “I identify as…” in combination with these categories: “woman”, “man”, “nonbinary”, “trans woman”, “trans man”, “genderqueer”, “genderfluid”, “agender” and “Two Spirit”. In addition, there was agreement that gender and gender identity should be regarded as separate entities.

These results, similar to previous studies (36, 47), highlight that providing gender diverse options in surveys allows for a more inclusive approach, acknowledging the existence and experiences of gender minorities. By offering categories beyond binary options, such as including non binary identities, surveys demonstrate inclusivity and enhance the accuracy of data collection by enabling individuals to state their gender identity more accurately. In addition, our results to assess both sex assigned at birth and gender (identity) regardless of the research question and as independent constructs, align with previous research such as the use of a two-item approach (32, 34) rather than a single, stand-alone sex and/or gender (identity) item (35).

Experts indicated that whether gender expression should be assessed depends on the research question. Consequently, unlike sex assigned at birth and gender (identity), gender expression may not always be relevant. There was no consensus on whether gender expression should be assessed categorically or dimensionally. However, if a dimensional assessment was preferred, there was a consensus to label the poles as feminine/masculine. Unfortunately, there was no consensus on which statement should be used to assess gender expression as the experts expressed varied preferences regarding a specific statement in both the first and second surveys with options such as “I identify as”, “I describe myself as”, and “I live as”. It was therefore only possible to provide recommendations for the answer categories (“genderfluid”, “androgynous”, “mostly masculine”, “mostly feminine”, “somewhat masculine”, “somewhat feminine”, “neither feminine nor masculine”).

The difficulties experienced by the authors in reaching a consensus regarding aspects of gender expression could have been due to several reasons: on the one hand, the lack of power from only 17 experts, and on the other hand, the relatively limited attention towards the measurement of gender expression in current research, which predominantly concentrates on surveying gender (identity) (39). In addition, the existing APA definitions do not seem to have been comprehensible and/or appropriate, as we were able to achieve a high level of consensus after revising these definitions. Consensus on the more tangible construct of sex assigned at birth was significantly higher than for gender (identity) and gender expression. We believe that this could indicate uncertainties with these constructs and highlight the need to formulate recommendations for data collection. Moreover, we found that studies that encompass the concepts of sex assigned at birth and gender (identity) should also survey sexual orientation, romantic orientation, and sexual behaviors. At the same time, while the representation of diversity aspects is important, we must acknowledge that assessments for research purposes need to be manageable (i.e., feasible in terms of study procedures) and to allow solid analyses (in terms of adequate statistical power for the groups under investigation). This could be another reason why aspects other than sex assigned at birth have not been included in a far-reaching manner in research so far.

It might also be relevant to include other aspects in the survey of gender expression such as the perception by others. For example, Wylie et al. (54) and The GenIUSS Group (36) suggested a two-item measure and included the question of how others would rate appearance, style, or clothing. Furthermore, there are suggestions for an additional extension with unipolar response scales (36, 39), which is consistent with our recommendations. Future research should aim to describe evidence gaps and ensure better representativeness of results by including gender specific populations. However, as already emphasized, it is also important to consider gender conformity/non conformity.

In terms of intersectionality, there are also several other aspects besides sex and/or gender impacting study applicability and results (3, 5, 14–16, 55, 56) most likely in a potentized, not always disambiguate way. These aspects, such as cultural background or ethnicity (e.g., 57, 58), significantly impact the mental health and well-being of individuals and should therefore be considered in research independently of the results presented here.

In contrast to other recommendations, such as the “Sex and Gender Equity in Research” (SAGER) guidelines (19), which primarily serve to standardize the reporting of sex and gender in research articles, our study addressed the preceding issue by focusing on the comprehensive assessment of these concepts. Recently, Stadler et al. (47) proposed a “Diversity Minimal Item Set” (DiMIS) to help close the diversity and gender gap. Concerning the concepts of sex assigned at birth and gender identity, the authors recommended using a single item which they adapted from the NHS England and LGBT Foundation (59), offering a range of gender diverse options, such as “nonbinary”, “trans” and “questioning”, consistent with our recommendations. In addition, the authors recommended to always provide the opportunity to self-identify; the possibility to opt out by including an option like “prefer not to answer” should also be considered.

In contrast to our study, existing guidelines did not include a recommendation for the assessment of gender expression. As such, we were also able to present consented definitions of the constructs. Additionally, our research emphasizes the integration of gender diverse populations into studies, a dimension that the “Sex and Gender Equity in Research” (SAGER) guidelines do not explicitly address. Therefore, our work adds a new perspective on including and assessing gender diverse populations. This aligns with other research that contends that including measures of gender expression in survey research is crucial for capturing the diverse ways in which gender is understood and experienced and in which gender inequality affects opportunities in life (40).

### 4.2 Limitations

Although this was the first project questioning international experts in the field of gender diversity on the assessment of sex, gender, and gender expression, we could only reach 17 experts from a total of four countries. The response rate in survey one was 26%, in survey two 50%. Although this may not seem like a large number at first, other expert studies show a similar number of participants (e.g., 53, 60). Nonetheless, the results should be interpreted with caution in view of the relatively small sample.

Another limitation concerns the background diversity of experts, as most were from either Canada or the United States of America. This is the result of our expert selection method, as only first and last authors mentioned in thematically relevant publications were included, in addition to the fact that not all experts accepted the invitation to participate in the study. As a result, it has not been possible to include the perspective of other countries in a sizeable part of the world, even though their perspectives would have most certainly been enriching. Therefore, our findings may not necessarily be suitable for all/further cultures and continental contexts. Also, vocabulary and use of the concepts under investigation may differ in diverse cultures or languages and may not be transferable, but experts were asked to give examples of alternative vocabulary in their respective native language – although no answers were given here. The 70% consensus threshold was determined arbitrarily; nevertheless, other studies have successfully employed the same cut-off point (51–53).

Of course, it is also a limitation that the suggestions presented here derived from researchers and have not yet been harmonized with LGBTQI+ community members, patients, or other key stakeholders, which would have provided valuable input for the formulation of practical recommendations. However, it is particularly important to emphasize that eleven out of 16 experts stated that they were part of diverse communities themselves.

## 5 Conclusion

This study offers definitions for sex assigned at birth, gender (identity), and gender expression that have been refined with input from experts, along with new diversity-oriented recommendations for clinical studies. By clarifying the definitions of the constructs, we aim to promote their more precise use in future clinical research. Standardized surveys could facilitate better comparisons of results and ensure that gender is recognized beyond binary expressions in clinical trials to improve the healthcare of SGM individuals.

## Data Availability

The data that support the findings of this study are available from the corresponding author upon reasonable request.
