# Supplementary material for: An expert perspective on diversity-oriented standards for assessing sex and gender in clinical research
Source: Front Psychiatry. 2025 Jan 29;15:1448487. doi: 10.3389/fpsyt.2024.1448487 (PMC11814446; doi:10.3389/fpsyt.2024.1448487)
Supplement: Supplementary file 1 [file SupplementaryFile1.docx]

Supplementary Material

Expert-based recommendations on diversity oriented standards to assess sex and gender in clinical research

Hannah R. Hambruch^1^, Nora M. Laskowski^2*^, Robert-Paul Juster^1,2^, Georg Halbeisen^1^, Georgios Paslakis^1^

^1^ University Clinic for Psychosomatic Medicine and Psychotherapy, Medical Faculty, Campus East-Westphalia, Ruhr-University Bochum, Luebbecke, Germany

^2^ Research Center of the Institut Universitaire en Santé Mentale de Montréal, Department of Psychiatry and Addiction, Université de Montréal

# Supplementary Figures and Tables

Supplementary Table 1: Possible umbrella terms

|  | **Answer Scale** | |
| --- | --- | --- |
| **Terms** | **Yes**  *n* (%) | **No**  *n* (%) |
| Nonbinary | 6 (60.0) | 4 (40.0) |
| Genderqueer | 6 (60.0) | 4 (40.0) |
| Genderfluid | 3 (30.0) | 7 (70.0) |
| Agender | 2 (20.0) | 8 (80.0) |
| Diverse | 4 (40.0) | 6 (60.0) |
| Sometimes male, sometimes female | 1 (10.0) | 9 (90.0) |
| No identification | 8 (80.0) | 2 (20.0) |

*Note. The terms in bold achieved a consensus of >70%.*

Supplementary Table 2: Diversity measures

| **Measure** | **Answer Scale** | | |
| --- | --- | --- | --- |
|  | **Known and used** | **Known but not used** | **Not known** |
| 2-step Gender Identity Measure (1) | 5 (29.4) | 7 (41.2) | 5 (29.4) |
| Multidimensional Test Measure (2) | 3 (17.6) | 10 (58.8) | 4 (23.5) |
| Gender Identity Scale (3) | 2 (11.8) | 7 (41.2) | 8 (47.1) |

# References

1. Reisner SL, Biello K, Rosenberger JG, Austin SB, Haneuse S, Perez-Brumer A, Novak DS, Mimiaga MJ. Using a Two-Step Method to Measure Transgender Identity in Latin America/the Caribbean, Portugal, and Spain. *Arch Sex Behav* (2014) 43:1503–1514. doi: 10.1007/s10508-014-0314-2

2. Bauer GR, Braimoh J, Scheim AI, Dharma C. Transgender-inclusive measures of sex/gender for population surveys: Mixedmethods evaluation and recommendations. *PLoS One* (2017) 12: doi: 10.1371/journal.pone.0178043

3. Ho F, Mussap AJ. The Gender Identity Scale: Adapting the Gender Unicorn to measure gender identity. *Psychol Sex Orientat Gend Divers* (2019) 6:217–231. doi: 10.1037/sgd0000322
